# How proteins open fusion pores: insights from molecular simulations

**DOI:** 10.1007/s00249-020-01484-3

**Published:** 2020-12-19

**Authors:** H. Jelger Risselada, Helmut Grubmüller

**Affiliations:** 1 Department of Theoretical Physics, Georg-August University of Göttingen, Göttingen, Germany; 2 Leiden University, Leiden Institute of Chemistry (LIC), Leiden, The Netherlands; 3 Max Planck Institute for Biophysical Chemistry, Theoretical and Computational Biophysics Department, Göttingen, Germany

**Keywords:** Toroidal, Stalk, Pore, Nano dics, Free energy

## Abstract

Fusion proteins can play a versatile and involved role during all stages of the fusion reaction. Their roles go far beyond forcing the opposing membranes into close proximity to drive stalk formation and fusion. Molecular simulations have played a central role in providing a molecular understanding of how fusion proteins actively overcome the free energy barriers of the fusion reaction up to the expansion of the fusion pore. Unexpectedly, molecular simulations have revealed a preference of the biological fusion reaction to proceed through asymmetric pathways resulting in the formation of, e.g., a stalk-hole complex, rim-pore, or vertex pore. Force-field based molecular simulations are now able to directly resolve the minimum free-energy path in protein-mediated fusion as well as quantifying the free energies of formed reaction intermediates. Ongoing developments in Graphics Processing Units (GPUs), free energy calculations, and coarse-grained force-fields will soon gain additional insights into the diverse roles of fusion proteins.

## Introduction

Membrane fusion, one of the most fundamental processes in life, occurs when two separate lipid membranes merge into a single continuous bilayer. In the last decades, much understanding has been gained about the intermediate steps of the biological fusion reaction due to extensive theoretical and experimental research. It has now become widely accepted that the fusion reaction must proceed through a hemifusion state before the fusion reaction can progress into the formation of a fusion pore (Chernomordik and Kozlov 2005, 2008; Risselada and Mayer 2020).

**Fig. 1:**
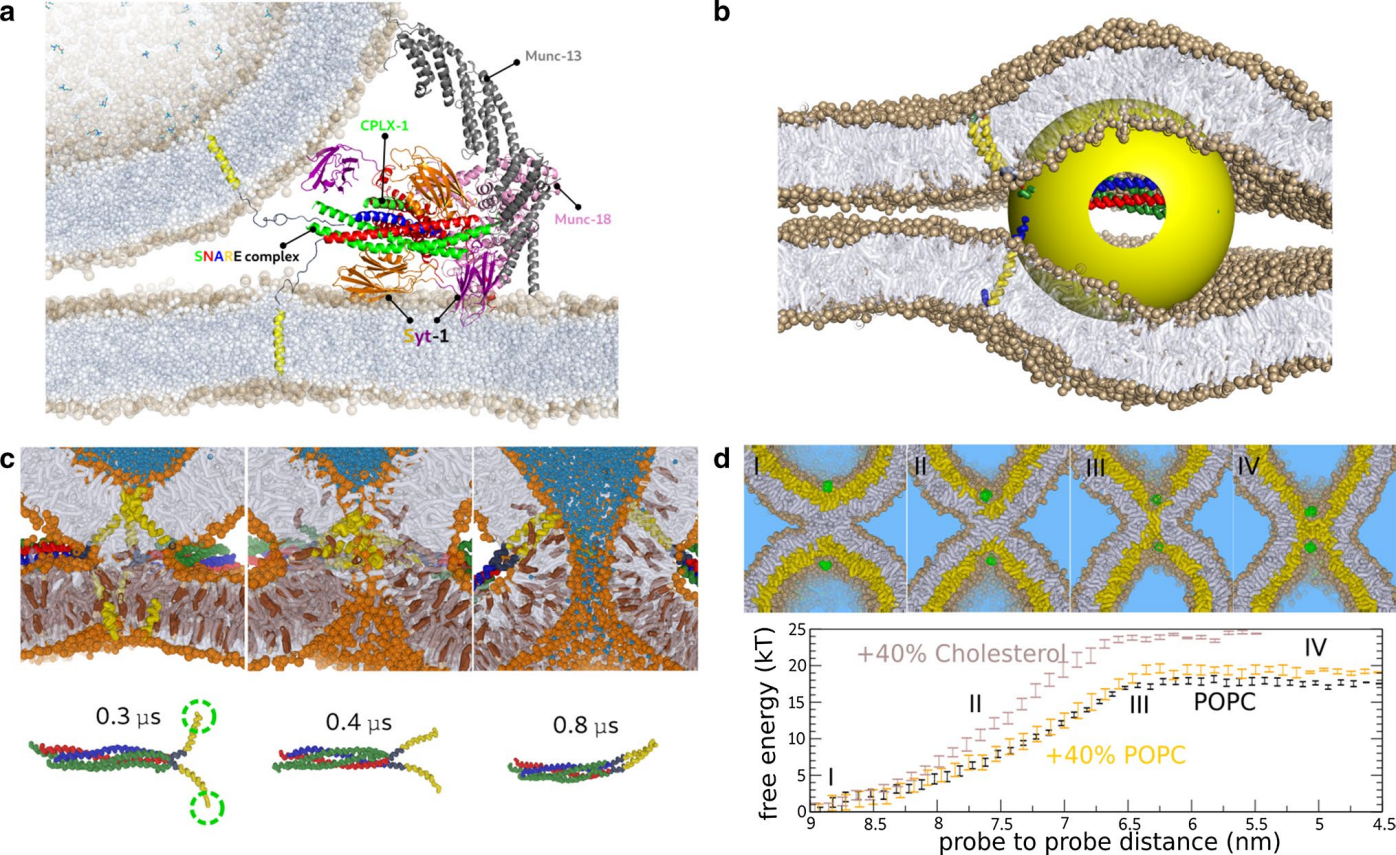
Molecular simulations of the molecular fusion engine. (a) Molecular model of the complete synaptic fusion machinery (adapted from Ref. (Bassereau 2018)). (b) The yeast fusion machinery as a model of the SNARE-mediated inter-cellular fusion machinery (adapted from Ref. (D’Agostino et al. 2018)). The yellow sphere models the head of the SNARE-associated HOPS complex. The volume of the HOPS complex poses a steric repulsion on the fusion site in the low curvature regime. (c) SNARE-mediated expansion of the stalk into a fusion pore. The green circles indicate the hydrophilic ends of the transmembrane domains. (d) Free energy cost associated with the widening of the stalk when modeling the action of SNARE complexes. The plateau region is related to the formation of a critical transmembrane contact (the barrier). At this stage, subsequent expansion occurs in the absence of additional work

Notwithstanding this progress, many open and newly emerging questions remain regarding the nature of the rate-limiting steps of the fusion reaction and how the many participating fusion proteins such as, for example, SNARE molecules and associated tether proteins (e.g., see Fig 1a) help overcoming the free-energy barriers along the transition path (Smirnova et al. 2019). Furthermore, since membrane fusion is an essential step in the infection process of enveloped viruses such as, for example, pandemic viruses (i.e., Influenza and Corona viral strains), gaining further understanding of membrane fusion is ultimately driven by the (urgent) need to control it. In fact, the ability to predict how molecules alter the free energy landscape of membrane remodeling may have far-reaching applications in medicine and pharmacology, because it enables the rational design of novel fusion inhibitors (e.g., antivirals) or accelerators (e.g., gene transfection and drug delivery). Moreover, as we will discuss within this review, unraveling the underlying free energy landscape of the fusion reaction via molecular simulations also yielded critical new insights into the diverse roles of the viral, neuronal, and cellular fusion machinery.

Understanding the roles and mechanisms of fusion proteins is intimately connected to the free energy of the intermediates formed during the fusion reaction. However, theoretically quantifying free energies of fusion intermediates poses a major challenge because the essential reaction intermediates in biological membrane fusion are complex and of a molecular length scale. Traditional equation-based free-energy descriptions, such as Helfrich elastic continuum modeling, must coarse-grain the effects of lipids and proteins into a few parameters, which mathematically describe the molecular shape (spontaneous curvature) and the elastic moduli of the membrane (e.g., Ref. Ryham et al. 2016). While continuum elastic models can provide intuitive physical insights into the universal aspects of the free-energy landscape, they lack molecular details that may become important, particularly for the primary fusion steps and protein-mediated fusion. Furthermore, continuum elastic models impose radial symmetry to solve the associated shape equations of lipidic fusion structures. However, as we will discuss in this review, a compelling amount of evidence from molecular simulations and experiments has indicated that known lipidic fusion structures such as the stalk and fusion pore often break their radial symmetry to lower their free energy.

In molecular dynamics simulations, the physical driving forces that govern the reaction paths of non-chemical biological reactions are approximated by molecular force fields, composed of a set of functions and parameters that describe the different types of forces between atoms or coarse-grained groups of atoms. Therefore, molecular dynamics simulations, in principle, enable an explicit description of the full molecular complexity of biological membrane fusion. However, employing molecular force fields for the quantification of free energies is severely limited by the computational sampling of all accessible states of a physical system (phase-space).

During the last decade, computational sampling of phase-space has benefited from the development of faster CPUs and in particular graphics accelerators (GPUs). Also, from the side of molecular modeling, a speed-up by a factor of two hundred in comparison to atomistic simulations has been obtained by employing so-called coarse-grained models such as, for example, the Martini model (Marrink et al. 2007), which map individual chemical groups into a single interaction site. Since these coarse-grained (CG) models capture the level of individual amino acids, they enable detailed simulation of fusion proteins (Fig. 1). However, secondary and ternary structure of fusion proteins must enter as a conserved model parameter because of the absence of explicit hydrogen bonding. Therefore, its limitation is that the protein structure has to be known and must remain relatively unchanged during the fusion reaction. A further limitation of coarse-grained models is that many atomistic degrees of freedom are eliminated and replaced by much fewer coarse-grained degrees of freedom, which changes their entropic contribution to free energies. As a result, while coarse grained force fields try to include those in an effective matter at room temperature, the resulting temperature dependence and heat capacities may be less accurate. Nevertheless, these models can at least phenomenologically capture the spontaneous assembly or ‘zipping’ of individual SNARE molecules (Risselada et al. 2011; Risselada and Grubmüller 2012), i.e., the main constituents in neuronal and cellular membrane fusion, into coiled-coil complexes (Fig. 1). It is noteworthy that the zipping of the SNAREs observed in these CG simulations extends into the membrane region, in agreement with the resolved X-ray structure of the fully assembled post-fusion state (Stein et al. 2009).

As we will discuss in more detail further below, significant methodological advances have been made in terms of free energy calculation and enhanced sampling methods. In particular, so-called ‘string methods’ display a large potential in resolving the free energy landscape of membrane fusion (Smirnova et al. 2019), since these methods self-resolve the reaction pathway of minimum free energy of the fusion reaction (e.g., the process of stalk formation between two apposed membranes)—quite in contrast to standard free energy calculations methods in molecular simulations which rely on an explicit description of a likely pathway, which may or may not be a minimum free energy pathway.

The goal of this review is twofold: (i) To present insights into how fusion proteins and other external enhancers of membrane fusion (e.g., electric fields (Haluska et al. 2006), functionalized nano-particles (Tahir et al. 2020), and carbon nano-tubes (Bhaskara et al. 2017)) can actively alter or overcome fusion barriers based on the insights of molecular simulations. (ii) Providing a set of ‘lessons learned’ concerning the many pitfalls and challenges that one may encounter when studying membrane fusion by molecular simulations.

We will chime in at the stage where an initial fusion stalk has already formed and discuss how its subsequent evolution into a fusion pore can be actively driven. One of the main foci of this review is to discuss the role of asymmetry of the fusion site in reducing the free energy of both the stalk and fusion pore. Hence, quite in contrast to earlier elastic continuum models, molecular simulations are free of an implicit symmetry bias when studying the evolution of the fusion reaction and subsequently formed fusion pore. We highlight these diverse roles of asymmetry together with its corresponding microscopic observation. Finally, we will discuss the state-of-the-art in resolving the free energy landscape of protein-mediated membrane fusion via molecular simulations.

### Expansion of the stalk in SNARE-mediated fusion

The free energy barrier against stalk expansion, often coined ‘radial stalk expansion’, is characterized by the formation of an initial contact between the transleaflets—a critical transleaflet contact. Because the expansion of the stalk does not necessarily proceed radially (see Fig. S4 in Ref. (Risselada et al. 2014)), we address this reaction pathway via the collective term ‘stalk widening’. SNARE complexes can actively overcome the barrier against stalk widening by mechanically imposing local pressure on the transleaflets via the hydrophilic ends of the transmembrane domains (TMDs), which facilitates local indentation of the transleaflets. In essence, this mechanism relies on an elastic force balance between the force associated with a collective growth of stalk area and the point-like force imposed by the TMD ends on the transleaflets.

It is noteworthy that the local chemical nature of the TMD ends can, to some extent, affect the (local) free energy cost required to indent the transleaflets and form a critical transleaflet contact via active perturbation (Wehland et al. 2016; Sharma and Lindau 2018). Interestingly, stalk widening can be alternatively driven by placing a 12 nm-sized open carbon nano-tube (CNT) through the stalk’s hydrophobic core, which bridges both transleaflet of the stalk (Bhaskara et al. 2017). The free ends of open carbon nano-tubes are oxidized and hydrophilic of nature, similar to the ends of SNARE TMDs. As the CNT favorably tilts away from the symmetry axis connecting the vesicle centers, the transleaflets are forced to merge (Bhaskara et al. 2017).

If fusion occurs between two already highly curved membranes, then ‘fusiogenic’ lipids with negative spontaneous curvature (e.g, POPE and cholesterol) are not expected to further lower the expansion barrier within this mechanism (Risselada et al. 2014), in contrast to the fusion reaction at low membrane curvature (D’Agostino et al. 2017). This discrepancy may be explained by the fact that the hydration of lipid headgroups at the circumference of the corresponding ‘dimple stalk’ is actually larger than that of the corresponding lipid in a fully hydrated flat membrane—the stalk is effectively not a negatively curved structure compared to a flat membrane (see Fig. S6 in Ref. (Risselada et al. 2014)). Stalk widening in dimple fusion (high membrane curvature) seems thus not limited by costly dehydration of lipid headgroups, but rather by relative stretching of the transleaflets to form an initial contact. This explains why cholesterol can oppose stalk widening in dimple fusion since it increases the elastic moduli of the leaflets (Risselada et al. 2014). In contrast, the presence of unsaturated lipids is therefore expected to enhance stalk widening since these lipids reduce the elastic moduli. Perhaps this is one of the many reasons why the presynaptic plasma membrane is highly enriched in poly unsaturated lipids (Takamori 2006), especially when stalk expansion is the rate-limiting step in the fusion reaction (Baoukina and Tieleman 2010; Kawamoto et al. 2015; Risselada and Grubmüller 2012; Bruininks et al. 2020). Notably, a later study using the Shinoda-DeVane-Klein (SDK) coarse-grained model did find an additional reduction of the expansion barrier in small vesicles when (unsaturated) DOPE lipids were added to a vesicle consisting of saturated DMPC lipids (Kawamoto et al. 2015). The question is to which extend this effect relates to DOPE’s unsaturated tails rather than its PE head group (both variables were changed simultaneously).

At low membrane curvature, where the protein-free barrier against stalk widening is in the range of 100 $k_BT$ or more (Kawamoto et al. 2015), active widening of the stalk posses a severe challenge. Even the presence of three Yeast SNARE complexes at the fusion site still yields a residual barrier of about 70 $k_BT$ against expansion of the stalk (D’Agostino et al. 2017). Fusion within the low curvature regime may thus rely on the local generation of high membrane curvature (Risselada and Mayer 2020). Interestingly, the additional presence of a single voluminous SNARE-associated tether complex can already reduce the free energy barrier against stalk widening to about 35 $k_BT$ by imposing steric repulsions on the site of hemifusion (Fig. 1). These repulsions yield an asymmetrically perturbed or curved fusion site. The adopted asymmetry at the fusion site generates a stalk structure that is closer to the native structure of the critical intermediate (the free energy barrier) at low membrane curvature (D’Agostino et al. 2018). *In vivo*, these tether complexes likely enforce a peripheral location of the SNARE complex near the curved membrane edge (the vertex) of the contact/adhesion zone formed between large vesicles (Risselada and Mayer 2020).

Finally, since the formation of a critical transleaflet mainly relies on the ability of the transleaflet to stretch, the resultant barrier against stalk widening is extremely susceptible to the lipid distribution between cis- and transleaflet (Risselada et al. 2014). Even slight variations in the lipid distribution between the monolayers (a few percent only) will already give rise to a substantial interleaflet tension, which can change the free energy barrier by several tens of $k_BT$. Since the time scale of molecular simulations does not suffice to achieve equilibrium between the leaflets via spontaneous lipid flip-flops, the apparent stalk expansion barrier will, therefore, strongly depend on the construction of the initial membrane setup. However, equilibrium between the monolayers can be alternatively ensured and tested for using ’artificial membrane pores’, which enable high lipid flip-flop rates within simulation time scales (Risselada and Marrink 2009). We emphasize that changing membrane composition in an equilibrated vesicle by redefining interaction types (a trivial procedure in coarse-grained models), will also introduce interleaflet tension, since each membrane composition has a unique lipid distribution between the inner and outer monolayer at equilibrium (Risselada and Marrink 2009).

Special care is also required when studying fusion with asymmetric planar membranes whose leaflets have different lipid compositions. These are typically constructed by ‘leaflet area’ matching, i.e., the area of each leaflet is estimated by simulating the corresponding symmetric membrane. However, it is important to emphasize that ‘leaflet area matching’ does not guarantee vanishing interleaflet tension since the two corresponding parental symmetric membranes usually have rather different pressure profiles. In contrast, ‘artificial pores’ likely enable a rapid net material exchange between the leaflets without significant loss of compositional membrane asymmetry.

Furthermore, the presence of sterols or other small hydrophobic molecules with a high transversale diffusion rate likely can reduce interleaflet tension considerably, because they alternatively enable equilibration of the chemical potential between the two leaflets. Because generation of interleaflet tension offers an extremely effective mechanism to drive the widening of the stalk, it is tempting to think that such a mechanism may be exploited by the fusion machinery *in vivo*. For example, by inducing electrostatic condensation within the cisleaflets (e.g., anionic lipid in the presence of calcium) (Tahir et al. 2020). However, such a mechanism would require a high level of regulation (calcium influx/efflux), and dissipation of the induced transleaflet tension must be restricted on the timescale of the fusion reaction.

Whether stalk widening ‘escapes’ into forming a stable hemifusion diaphragm (HD) rather than a fusion pore likely depends on inter-membrane distance and the concomitant curvature of the formed ‘stalk wings’ (Nishizawa and Nishizawa 2013), lipid composition (Risselada et al. 2014; Gardner and Abrams 2017), and the number of surrounding SNARE complexes present (Risselada et al. 2011). In protein-free dimple fusion of POPC membranes (curvature of 1/8 $\hbox {nm}^{-1}$), the barrier against HD rupture is 1–2 $k_BT$ and therefore negligible. This is because HD growth is limited to a size of 2 nm, which results in the formation of an extremely thin and unstable connecting membrane. However, addition of 30% cholesterol increases the barrier against HD rupture to about 10 $k_BT$ (see Fig. S3 in Ref. (Risselada et al. 2014)) (García et al. 2001; Risselada et al. 2014).

Notably, the reduced thickness of a small HD is not explained by the presence of excess membrane tension. This notion is supported by the observed reduction of fusion pore size when HD material is absorbed, indicating that the thinner HD is not under an excess tension (see Fig. 3A and Ref. (Risselada et al. 2014)). Instead, the reduced membrane thickness is a consequence of the material equilibrium (lipids can freely exchange) between the flat membrane part (the HD) and the curved membrane leaflet (the tri-junction or rim of the HD). The chemical potential of a curved leaflet is substantially larger than that of a flat, non-curved leaflet (Grafmüller et al. 2013). The HD compensates for the increased chemical potential at its rim by reducing the thickness of its central membrane (Grafmüller et al. 2013). In essence, the pronounced thinning of a small HD thus results from the absence of bulk properties. These finite size effects fade when the HD grows, increasing both its thickness and concomitant barrier against rupture (see Fig. S3 in Ref.(Risselada et al. 2014)). Artificially restricting the mobility of SNARE complexes surrounding the fusion site in molecular simulations destabilize HD formation by hindering its expansion towards a stable size (Sharma and Lindau 2018). The question is, of course, whether such a drastic spatial restriction of mobility, i.e., the usage of position restraints in molecular simulations, would occur also *in vivo*. Further, it is plausible that fusion facilitated by, e.g., a single SNARE complex (Mohrmann et al. 2010) could allow *in vivo* survival of an HD with interesting consequences for subsequent fusion pore formation (Fig. 3A).

### Expansion of the stalk in viral fusion

**Fig. 2:**
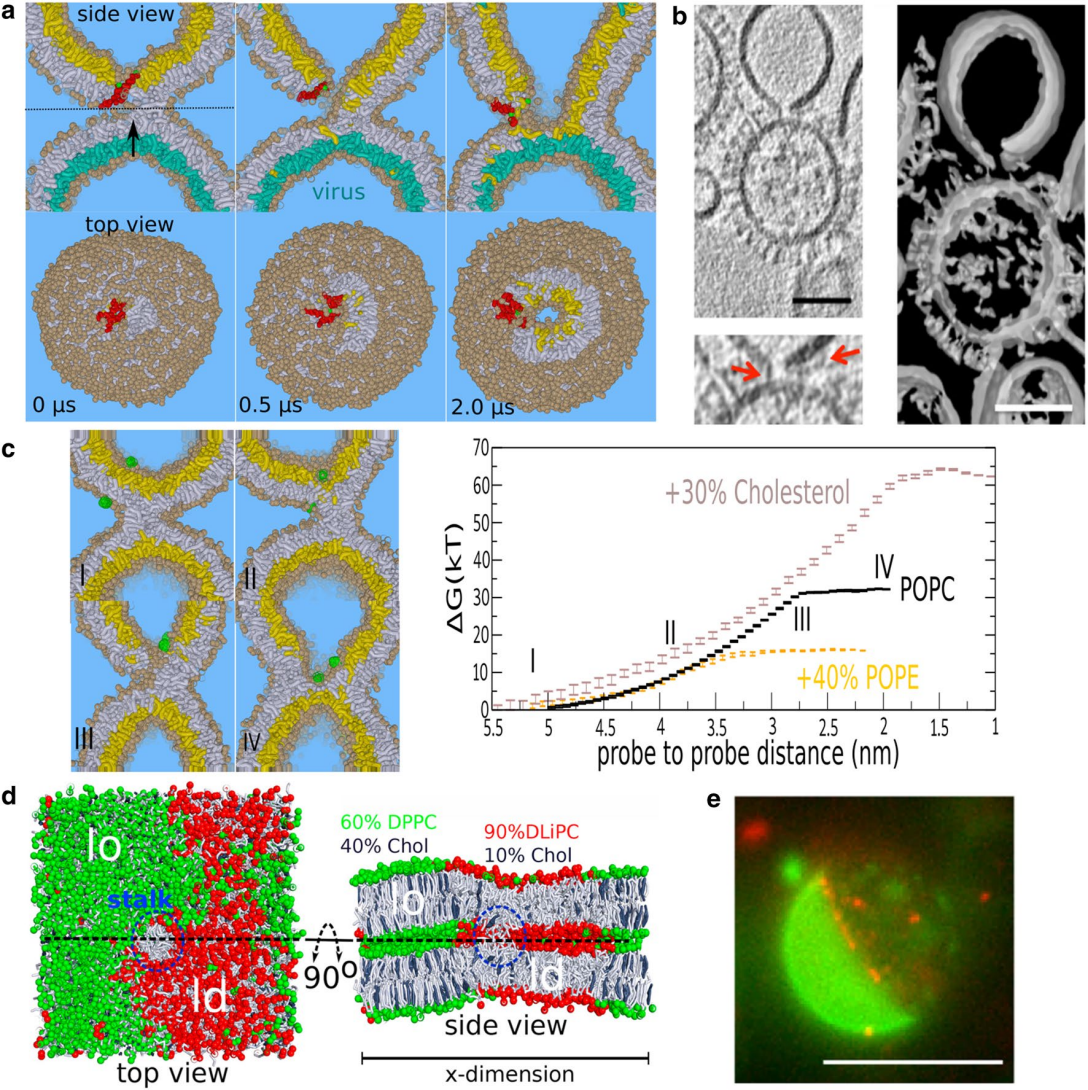
Asymmetric expansion of the fusion stalk. **a** Expansion of a stalk into a hemifusion diaphragm mediated by a nearby bundle of transmembrane oriented influenza fusion peptides. The presence of the peptides eases heterogeneous nucleation of a ‘bare hole’ in between the stalk and peptides. The free rim of the formed ‘hole’ is rapidly encircled by the stalk resulting in the alternative formation of an hemifusion diaphragm. **b** Electron cryotomography observations of modeled influenza fusion reveal the virus’ ability to form holes in the target membrane (a liposome) even in the absence of a lipidic connection. Adapted from Ref. (Chlanda et al. 2016). Scale bars: 50 nm. **c** Free energy associated with forming a stalk-hole complex (Risselada et al. 2014). The plateau region indicates the formation of a hole (the barrier), after which subsequent elongation of the stalk becomes spontaneous. **d** Fusion intermediates generally prefer to locate near interfaces: A fusion stalk binding to the lo/ld interface. **e** Modeled HIV fusion occurring at the lo/ld interface in experiments. Adapted from Ref. (Yang et al. 2016). Scale bar: 20 $\mu$m

Whereas SNAREs rely on a complementary partner waiting for them in the target membrane, viruses are pirates that must hijack cells. In contrast to SNARE complexes, viral fusion proteins are seemingly unable to exert direct mechanical force on *both* transleaflets of the stalk, because that would require their amphiphilic fusion peptides to first penetrate and fully span the host membrane (Risselada et al. 2014; Risselada and Grubmüller 2012), rather than residing on the surface of the host membrane (Skehel and Wiley 2000). Adaptation of such a transmembrane orientation is additionally linked to the rupture of the host membrane, as demonstrated by recent electron cryotomography observations of modeled Influenza fusion (Chlanda et al. 2016) (see Fig. 2b). Furthermore, if multiple fusion peptides adopt a transmembrane orientation in the direct vicinity of the stalk, widening of the stalk is vastly energetically outperformed by alternatively asymmetric stalk expansion mechanisms (see Fig. 2a) (Müller et al. 2003; Smeijers et al. 2006; Risselada et al. 2014). Quite in contrast to functional membrane fusion driven by SNARE complexes, it is thus not evident if or how the viral fusion machinery drives the active widening of the stalk. Could the viral fusion machinery actively expand a stalk by inducing favorable interleaflet tension in the target membrane? Such a mechanism is not evident, however, since the amphiphilic fusion peptides adhere to the cisleaflet of the target membrane and the concomitant creation of excess cisleaflet area would rather oppose widening of the stalk.

Whether an active mechanism to expand the stalk is required largely depends on the biological requirement of the rate of the fusion reaction. Obviously, viral fusion rate does not need a similarly fast rate as neuronal fusion (sub-millisecond). Free energy calculations have indicated the fusion reaction between a small highly curved 20-nm sized DMPC/DOPE (being 5 times smaller than most enveloped viruses) and a corresponding planar membrane already faces a substantial free energy barrier of about 50 $k_BT$ against non-leaky stalk widening (a non-leaky radial stalk expansion) (Kawamoto et al. 2015). This thus implies a considerable barrier against stalk widening and thus a high meta-stability of a formed stalk structure. Notably, most enveloped viruses are about five times larger, and thus have a much lower membrane curvature, and are additionally coated with a stiff matrix protein on its transleaflet. Both of these factors are expected to further increase the barrier substantially (Risselada et al. 2014; Kawamoto et al. 2015). Therefore, given that an active expansion mechanism is absent, and given that the mechanism would preferentially proceed via a non-leaky ‘radial’ stalk expansion, high-resolution electron cryotomography observations of viral fusion would abundantly reveal meta-stable stalk structures observed as small lipidic junctions between two fully intact membranes. However, such an intermediate has not yet been observed by high-resolution electron cryotomography of viral fusion (Chlanda et al. 2016). Therefore, the viral fusion machinery evidently involves an active and highly efficient mechanism to expand the stalk.

Interestingly, simulations suggest that a stalk can be alternatively expanded via nucleation of a ‘hole’ in the target membrane in the direct vicinity of the stalk (Fig. 2a,c). The barrier against stalk expansion within this asymmetric mechanism is the formation of the ‘hole’. Formation of a stalk-hole complex is favorable because of a mutual reduction of the free energy of both the stalk and the ‘hole’ (Müller et al. 2003; Katsov et al. 2006). Therefore, the presence of a metastable stalk inherently compromises the stability of the membrane against spontaneous pore formation (Müller et al. 2003; Katsov et al. 2006). As a consequence of the excess line tension present at the rim of the ‘hole’, the stalk rapidly encircles the hole forming a hemifusion diaphragm. Here, it is noteworthy to mention that interfaces are commonly seen to facilitate fusion reactions (e.g., see Fig. 2d,e). For example, modeled HIV fusion events preferentially occur at the boundary between liquid ordered/liquid disordered (lo/ld) domains (Yang et al. 2016) (see Fig. 2e). Indeed, the Ld/Lo interface can facilitate the attraction of fusion intermediates such as a fusion stalk (Risselada 2017) (see Fig. 2d) as well as amphiphilic (fusion) peptides (Su et al. 2020). Finally, also the excess line tension of the edge of a lipid nano disc can drive elongation of the stalk (Sharma and Lindau 2018).

The barrier against ‘hole’ formation in the stalk’s direct vicinity—this corresponds to the barrier against stalk expansion—is differentially affected by membrane composition (Risselada et al. 2014). Within such a mechanism, POPE lipids strongly decrease the barrier against stalk expansion, whereas cholesterol strongly increases it. In fact, in our simulations of dimple fusion, the ‘leaky’ expansion of the stalk is slightly favored over non-leaky stalk widening in the presence of POPE lipids (a free energy barrier of 15 $k_BT$ versus 17 $k_BT$, respectively). Interestingly, the free energy barrier within this ‘leaky’ mechanism is not affected by interleaflet tension (Risselada et al. 2014). Moreover, also restraining the bending of a transleaflet mimicking the membrane stiffening effect of viral matrix proteins does not affect the free energy barrier of his mechanism (Risselada et al. 2014) (Fig. 2a). The properties of such a mechanism are thus quite different from the properties of the stalk widening mechanism whose free energy barrier is highly sensitive to both changes in interleaflet tension and leaflet stiffness. Consequently, the external conditions at the site of membrane fusion can additionally determine which of these two *competitive* mechanisms is most likely. Because of the active force which SNARE proteins can directly exert on the transleaflets of a stalk structure as well as due to the abundance of sterols subject to high flip-flop rates (this reduces interleaflet tension), the SNARE fusion machinery can energetically outperform leaky fusion (Wang et al. 2009; Risselada and Grubmüller 2012).

Interestingly, our simulations illustrated that the presence of a metastable bundle in the target membrane formed by five to six trans-membrane oriented Influenza fusion peptides in the direct vicinity of stalk substantially eases heterogeneous nucleation of a ‘hole’ between the stalk and the peptide bundle (see Fig. 2a and Ref. (Risselada et al. 2014)). For such a peptide-facilitated mechanism, the presence of the peptide bundle effectively further weakens the membrane in the vicinity of the stalk, thus enabling the spontaneous formation of a ‘hole’ within simulation timescales. Since the collective adhesion of fusion peptides on the surface of the target membrane surface already substantially compromises membrane stability, stalk induced hole formation may in fact not require the presence of a nearby transmembrane bundle (Katsov et al. 2006). Thus, the viral fusion machinery can actively drive expansion of the stalk by exploiting the stalk’s native propensity to nucleate a ‘hole’ in its direct vicinity.

Furthermore, ‘puncturing’ of the target membrane in viral fusion precedes lipid mixing (Lee 2010; Chlanda et al. 2016). Such a phenomenon has recently been directly observed by electron cryotomography (Chlanda et al. 2016) (Fig. 2b). This mechanism somewhat resembles the experimentally observed mechanism of fusion driven by electroporation in the presence of an external electric field (Haluska et al. 2006; Böckmann et al. 2008). However, since the rim of such an isolated pore is likely coated by fusion peptides, thereby directly explaining its stability, subsequent elongation of a stalk formed at the rim of the pore would be severely hindered. Hence, both the stalk and the peptides compete for the excess line tension at the pore’s interface (Risselada et al. 2012). This scenario most likely explains the observation of intermediate structures featuring a large pore which is partially encircled by a stalk (coined ‘lipidic junctions’) (Chlanda et al. 2016). It is important to emphasize that the mechanism observed in simulations (Müller et al. 2003; Smeijers et al. 2006; Risselada et al. 2014) rather relies on the additional formation of a ‘bare hole’ in between the stalk and peptide bundle, and that the stalk subsequently competes with the peptides for the excess line tension of the hole’s rim by rapidly surrounding it.

The absence of a leaky viral fusion mechanism is often attributed to the alternative occurrence of a radial stalk expansion. Leakage in modeled Influenza fusion can be reduced but not entirely prevented by the addition of lipids with a negative spontaneous curvature to the host membrane (Chlanda et al. 2016; Haldar et al. 2018). In particular, the presence of 40% cholesterol effectively reduces the percentage of events with leaky phenotype (rupture events) to about 10% (Chlanda et al. 2016). However, this implies that the free energy barriers of the two different phenotypes nevertheless remain within about 2 $k_BT$ of each other. It is not evident how two competitive reactions can proceed via formation of both topologically and structurally different intermediates, yet remain energetically close over a large range of different conditions (Chlanda et al. 2016; Haldar et al. 2018). Intriguingly, also the presence of a single point mutation within the fusion peptide of Influenza (G13A) induces a predominantly leaky phenotype in the fusion reaction between red blood cells expressing Influenza Hemagglutinin (Lai and Tamm 2010). This observation indicates a subtle but direct involvement of the viral protein machinery itself to facilitate the leaky phenotype. It also demonstrates the propensity of the leaky reaction phenotype to occur even under *in vivo* membrane conditions. Molecular simulations revealed that the G13A mutant forms substantially larger metastable bundles or pores than the wild-type, thereby directly explaining its predominantly leaky phenotype (Risselada et al. 2012).

In contrast to electron cryotomography observations, the stalk-hole complexes observed in molecular simulations (Fig. 2a,c) feature an extremely small (size of about 1–2 nm) and transient hole (lifetimes of less than a microsecond). It is therefore quite unlikely that this reaction pathway would result in detectable leakage in experiments. Productive viral fusion may thus proceed via formation of a small ‘bare hole’ that is rapidly surrounded by a present stalk. This would explain how an apparently non-leaky phenotype remains tightly kinetically coupled to its leaky counterpart. Timing of stalk- versus hole formation likely controls the switch between the two phenotypes. Adding lipids with a negative spontaneous curvature enhances rapid stalk formation and thus competes with the observed ability to release stress by growing a large unproductive peptide coated pore (see Fig. 2b). In particular, the presence of cholesterol which additionally decreases membrane permeability (Bu et al. 2018) would delay excessive penetration of the fusion peptides and subsequent pore formation in the target membrane.

A direct consequence of a ‘leaky mechanism’ is an increased propensity to stabilize a hemifusion diaphragm (HD). Hence, in contrast to the mechanism of stalk widening, formation of the HD via ‘leaky fusion’ surpasses nucleation of an HD through a point-like intermediate, i.e., a transleaflet contact, and directly results in a larger HD of an already more stable size. The equilibrium size that an HD subsequently adopts is mainly a function of its membrane composition and does not necessarily reflect its pathway of formation. To this end, we hypothesize that viral fusion has an increased propensity for HD formation and that the viral fusion machinery must actively prevent its stabilization. This hypothesis is supported by the experimental observation that a single N-terminal point mutation within Influenza fusion peptides arrests the subsequent fusion reaction between viral particles and lipidic liposomes at the stage of an HD (Chlanda et al. 2016). It is noteworthy that a transmembrane bundle consisting of G1S mutants facilitates a stalk-hole mechanism with a similar effectivity as the wild-type in our dimple fusion setup (see Fig. 2a), even though its effectivity seemed reduced in planar membrane systems (Risselada et al. 2012).

Finally, recent multi-scale simulations studied the fusion reaction between a small vesicle coated by Influenza Hemagglutinin and a planar membrane (Pabis et al. 2020). Although the authors did not explore the nature of the stalk expansion mechanism in close detail (the snapshot in Fig. 2c in Ref. (Pabis et al. 2020) reveals a ‘leaky’ stalk elongation mechanism), they did observe the formation of a metastable HD after ‘widening’ of the stalk which eventually ruptured in the course of the simulations. The question is whether the proteins played a causal effect in overcoming these different free energy barriers and whether the observed mechanism corresponds to the actual mechanism *in vivo*. This could be tested by deleterious point mutations (e.g., G1S), analogous to tests performed in simulations of SNARE mediated fusion (Sharma and Lindau 2018).

### The asymmetric fusion pore

**Fig. 3:**
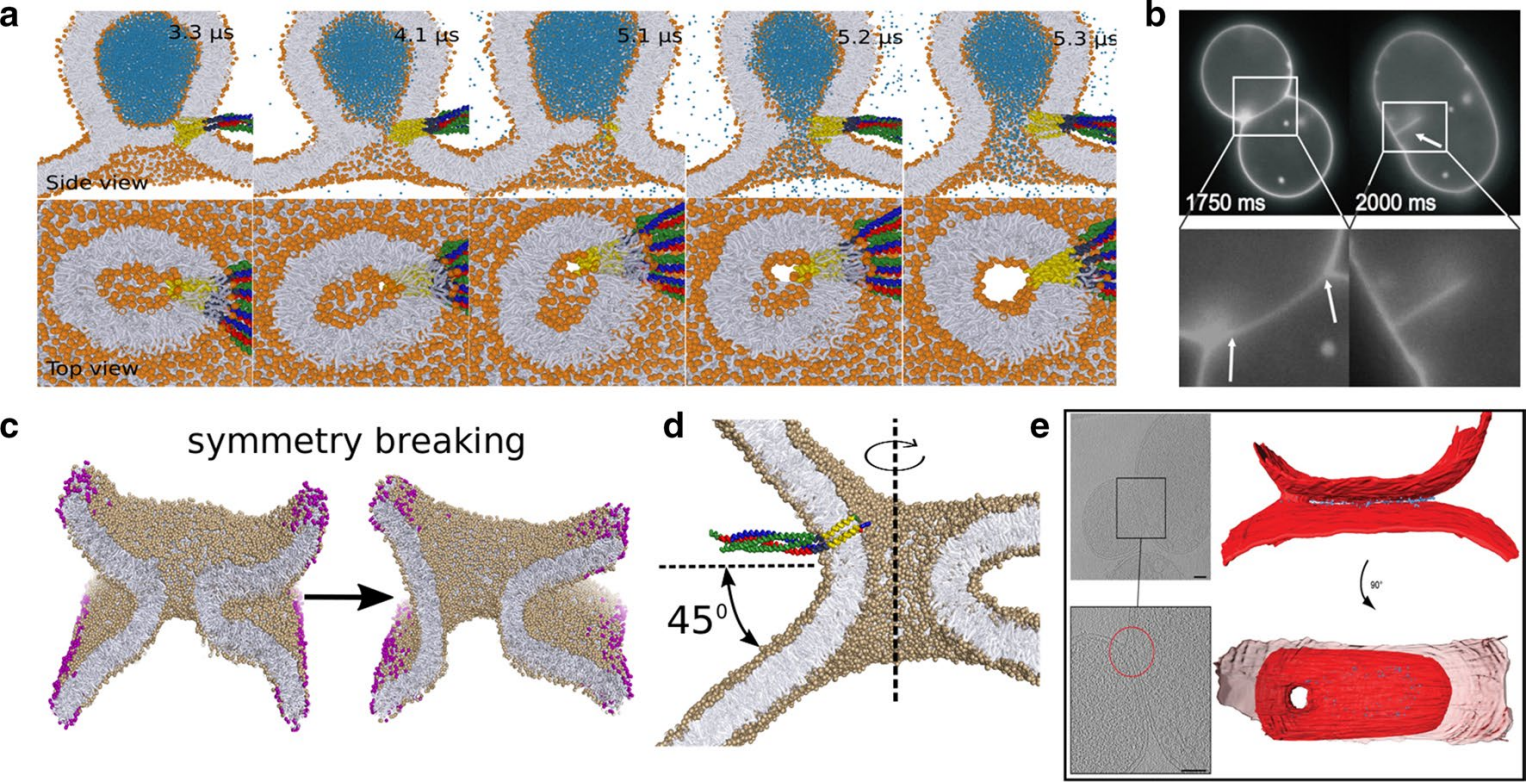
An asymmetric fusion pore. **a** SNARE-mediated formation of a pore at the rim of a hemifusion diaphragm. The pore displays a metastable flickering stage before it eventually expands into a toroidal fusion pore. **b** Direct microscopic observation of a rim-pore formed after rupture of an HD (adapted from Ref. (Nikolaus et al. 2010)). **c** A fusion pore centrally connecting two curved membrane sheets will favorably break its symmetry and expand to a larger equilibrium size. **d** The apparent local contact angle between the docked membranes defines the structural asymmetry and concomitant equilibrium size of the vertex pore. **e** Electron cryotomography observations of a vertex pore formed in modeled mitochondrial fusion (adapted from Ref. (Brandt et al. 2016))

The structure of the fusion pore is arguably one of the most central topics in the field of membrane fusion. It is widely accepted that fusion involves both lipids and integrated fusion proteins. The fusion pore is commonly assumed to be axially symmetric and toroidal. However, both molecular simulations and experiments have demonstrated that fusion pores preferentially adopt an asymmetric shape (Risselada et al. 2014; Blokhuis et al. 2020; Nikolaus et al. 2010; Brandt et al. 2016; D’Agostino et al. 2018; Grafmüller et al. 2007), with profound consequences for the free energy landscape of subsequent pore expansion (Risselada et al. 2014; Blokhuis et al. 2020). Two asymmetric types of fusion pores have been observed, (i) rim-pores formed at the rim of a single membrane thick hemifusion diaphragm (Gao et al. 2008; Nikolaus et al. 2010; Risselada et al. 2014) (see Fig. 3a,b), and (ii) vertex fusion pores formed at the edge or vertex of the contact zone formed during protein-mediated docking and adhesion of membranes (Grafmüller et al. 2007; Blokhuis et al. 2020; Brandt et al. 2016; D’Agostino et al. 2018) (see Fig. 3c,d,e).

*Rim-pores facilitate pore flickering.* Destabilization of a hemifusion diaphragm proceeds via formation of a pore formed at the rim of the hemifusion diaphragm: A rim pore. Similar to the expansion of a stalk, the ends of the SNARE TMDs exert a direct force on the rim of the HD which eases the heterogeneous nucleation of a rim-pore. Formation of a pore at such a location and concomitant symmetry breaking is favored because only part of the pore consists of the costly free membrane edge, whereas the other part involves the energetically less costly continuous membrane, i.e. the neck of a toroidal fusion pore. Because of its heterogeneous nature, the shape of such a pore is non-circular, and its overall shape during expansion is a direct consequence of free energy minimization (Risselada et al. 2014).

A rim-pore must expand up to a critical size (a free energy barrier), before complete expansion proceeds spontaneously (Risselada et al. 2014). Furthermore, the free energy landscape of rim-pore expansion up to the critical pore size can become rather shallow such that thermal fluctuations can facilitate substantial fluctuations in pore size and conductance (pore flickering) (Risselada et al. 2014) (see Fig. 3a). Such a pore flickering stage is followed by a sudden complete expansion if pore expansion surpasses the critical pore size. Similar to regular membrane pores, rim-pores can close in the absence of a (substantial) free energy barrier, especially in the absence of membrane tension. Therefore, so-called kiss-and-run events, i.e., transient opening and closing of fusion pores, in synaptic vesicles systems could be alternatively explained by the presence of rim-pores in small to medium-sized hemifusion diaphragms (a diameter of 10 nm or more) rather than by toroidal fusion pores. In particular, it is difficult to envision how closing of a toroidal fusion pore via hemifission events could occur in the absence of a GTP-driven fission machinery. The small, native fusion pores connecting yeast vacuoles can block the passage of soluble fluorescent dye molecules and consequently escape experimental detection in most fusion assays (D’Agostino et al. 2018). We therefore ironically coined these nanoscopic fusion pores ‘black holes’ (Risselada and Mayer 2020). Nevertheless, these small pores still enabled the passage of labeled transmembrane domains despite their charged C-termini, thereby illustrating a remaining ability to conduct ions (D’Agostino et al. 2018). However, complete blockage of ion conductance would require an even smaller pore size, less than 1 nm in diameter, and it is questionable if such a pore can be thermodynamically stable. In contrast, the formation and closing of rim-pores could result in transient conductance of ions. In that case, kiss-and-run behavior would be stimulated by the same factors that enhance the survival and growth of a small hemifusion diaphragm such as a reduced number of participating SNARE complexes at the site of membrane fusion and the presence of a high cholesterol concentration (Risselada et al. 2014). This may explain why cholesterol in some cases increases pore flickering (Wang et al. 2010) rather than reduces it (Stratton et al. 2016). This alternative explanation of kiss-and-run events has thus far been largely overlooked.

It is very likely that *in vivo* fusion pore formation competitively proceeds through both rim-pore formation and instantaneous toroidal fusion pore formation; the balance between these two pathways is rather subject to external conditions such as membrane composition and protein expression levels. Both of these pores, however, come with different pore flickering characteristics. In our simulations (Risselada and Grubmüller 2012), we observed that cholesterol laterally redistributes itself after toroidal pore formation, thereby reducing membrane asymmetry and driving additional opening of the fusion pore. However, the observed paradoxical ability of cholesterol to suppress pore opening and to facilitate pore flickering (Wang et al. 2010) is well explained by its competitive ability to stabilize an hemifusion diaphragm, which would eventually result in rim-pore formation (García et al. 2001; Risselada et al. 2014). Finally, in nano-disc based fusion assays, the toroidal fusion pore formed between a nano-disc and membrane features a volcano-like structure, which can subsequently collapse and seal into a continuous membrane because of the presence of excess line tension at the rim of the ‘volcano’ (Sharma and Lindau 2018). Such a pore collapse is an artifact of the usage of nano-discs and is not transferable to real membrane systems where such a line tension is absent. Therefore, comparisons of pore closing/opening dynamics (e.g., kiss-and-run events) between nano-discs and synaptic vesicles should be taken with care, as rather different factors may drive closing of the pore.

*The asymmetric vertex pore.* Recent experimental observations have illustrated that fusion pores between vesicles that are docked by an extended flat contact zone are located at the curved edge (vertex) of this zone (Brandt et al. 2016; D’Agostino et al. 2018) (e.g., see Fig. 3e). Using molecular simulations in conjunction with a wetting model, we have illustrated that a formed fusion pore experiences a direct attraction toward the vertex, because such a location enables the pore to lower its costly membrane bending energy (Blokhuis et al. 2020) (see Fig. 3c). A vertex pore likely characterizes the native structure of the fusion pore if the fusion reaction precedes through the formation of an extended contact zone between the membranes (e.g, endosomal fusion, mitochondrial fusion, yeast fusion, sperm fusion) (Blokhuis et al. 2020).

The size adopted by the resulting vertex pore strongly depends on the apparent contact angle between the adhered vesicles (see Fig. 3d), even in the absence of membrane surface tension. Larger contact angles substantially increase the equilibrium size of the vertex pore. Because the cellular membrane fusion machinery actively docks membranes, it facilitates a collective expansion of the contact zone and increases the contact angle. In this way, the fusion machinery can drive the expansion of the fusion pore by free energy equivalents of multiple tens of $k_BT$ (Blokhuis et al. 2020) from a distance and not only through the entropic repulsions exerted by the fusion proteins that reside within the fusion pore (Wu et al. 2017). Furthermore, also nano-discs are comprised of a peptide- or polymer-capped free membrane edge that introduces a spatially heterogeneous membrane environment analogous to the vertex of the docking zone. Because ‘edge attractions’ increase pore size and thereby likely affect measured pore conductance regardless of the edge’s structural nature (Blokhuis et al. 2020), it is an interesting question whether the fusion pore formed in experimental fusion assays based on larger nano-discs ($>20$ nm) preferably locates near the rim because of edge attractions or whether it adopts a central location because of edge repulsions.

### Free energy calculations of fusion intermediates

To quantify the kinetics of membrane transformations, one needs to study reaction paths and the free-energy barriers along these paths. However, using molecular force fields to quantify free energies is severely limited by the need to sample all accessible states of a physical system (phase-space). Furthermore, for collective processes involving many different molecules, such as membrane fusion or fission, it is often rather unclear how the reaction path should look like, i.e., how to define a suitable reaction coordinate which uniquely describes states along the fusion reaction, and how to control it. A related question is whether an *a priori* chosen reaction path actually represents a low free energy and reversible reaction path, since the presence of hysteresis compromises the accuracy of the free energy calculation. Below we will briefly outline and discuss the different approaches that have thus far been employed to access the free energy landscape of membrane fusion.

*Markov state modeling* has been successfully used (de Groot et al. 2001; Noé et al. 2007) to classify and identify different metastable intermediates states within the fusion reaction and to quantify their connecting reaction rates (Kasson et al. 2006). This approach, however, requires excessive sampling. Further, the (unbiased) barrier of the fusion reaction must be accessible within the simulation time-scales (Kasson et al. 2006). The advantage of this approach that direct information of the actual rate is obtained and thus the kinetic prefactor of the reaction rather than only the free energy barrier. It is important to emphasize that although such an approach involves no direct control of the fusion reaction (unbiased sampling), the obtained reaction rates still necessarily depend on the definition of the reaction coordinate.

*Tension interpolation methods* rely on the fact that an externally applied tension can enhance the fusion reaction such that it frequently occurs within the time-scales of molecular simulations (Shillcock and Lipowsky 2005; Grafmüller et al. 2009). This method relies on simply counting the number of occurred fusion events observed within large multiples of performed simulation at different levels of applied tensions. The number of events at a given tension is subsequently interpolated to the corresponding situation at zero tension.

*Forward flux sampling*. These methods define a series of interfaces between the initial and final states to calculate rate constants and resolve transition paths for rare events (Borrero and Escobedo 2007; Allen et al. 2009). These methods require no active force to enhance sampling—in contrast to umbrella sampling methods. Instead, sampling can be alternatively enhanced via statistical biasing by starting simulations (trial runs) from states characterized by a forward progress along an defined reaction coordinate. These methods may have an advantage in collective, diffusive processes such as, for example, membrane fusion where the relationship between a defined reaction coordinate and the concomitant umbrella forces are not trivial (Bubnis and Risselada 2016). In addition, they gain additional insight into the actual rate of the reaction (kinetic prefactor). Nevertheless, these methods become less practical if the free energy barriers are large (e.g., multiple tens of $k_BT$) since that would require (i) an large number of trial runs to drive the reaction forward, and (ii) reconstruction of rates via post analysis requires lots of data storage.

**Fig. 4:**
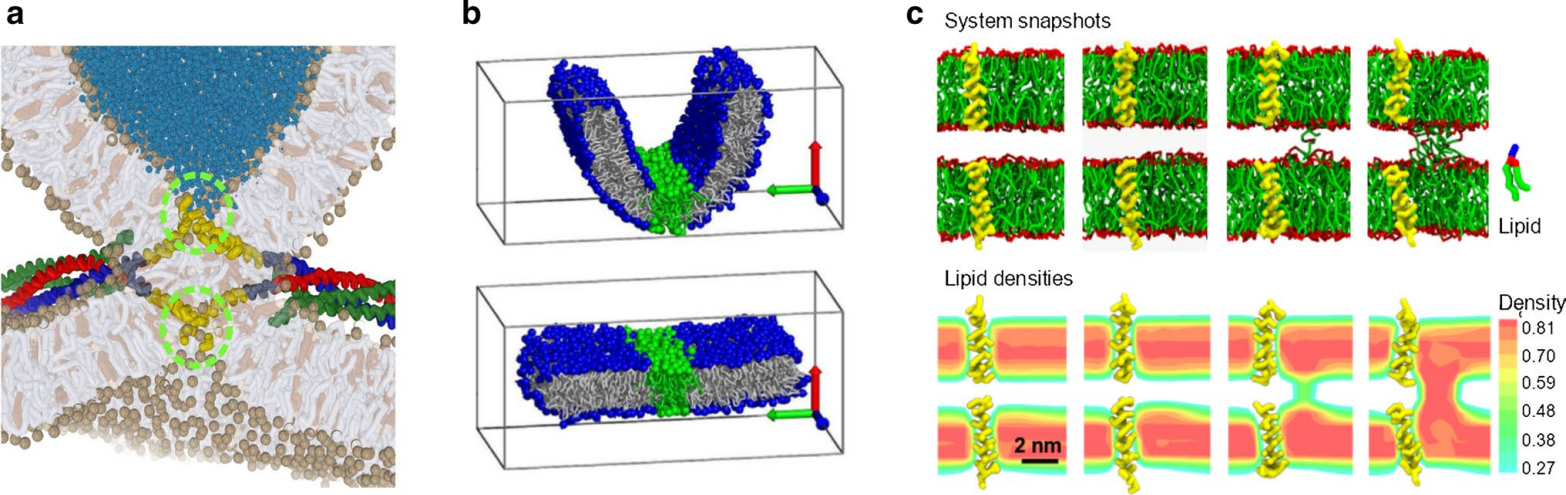
Examples of methods and reaction coordinates used to probe the free energy landscape of membrane fusion. **a** Reaction coordinates based on external local perturbation of the membranes (Risselada et al. 2014). For example, the hydrophilic ends of transmembrane domains are actively pulled within closer vicinity, thereby mimicking the action of SNARE complexes when being subject to unlikely thermal fluctuations. **b** Permutation reduction and collective coordinates enable the extraction of global modes within diffusive lipid membranes. The example illustrates the enforced bending and unbending of a membrane sheet, revealing deviations from the linear Helfrich behavior at high curvatures. The green-colored lipids illustrate the effective removal of lipid diffusion during membrane unbending (Bubnis and Risselada 2016). **c** The coupling of local membrane structure to local hydrophobic density in conjunction with a string method can self-resolve the reaction pathway of minimum free energy between two end states of interest. In this example, the process of stalk formation mediated by isolated transmembrane domains of SNARE complexes is studied. (Smirnova et al. 2019)

*Local perturbation* is a straight-forward method to estimate the free energy barriers within the stalk-pore transition. In contrast to the above approaches, here an active force is exerted on the transleaflet of the stalk by bringing two enlarged hydrophilic beads or lipids (Risselada et al. 2014), or the C-termini of the SNARE TMD (Sharma and Lindau 2018) within close proximity until barrier crossing spontaneously occurs within the microsecond time scale of the simulation (Fig. 4a). This approach requires that the residual barrier of the perturbed state is small, a few $k{_B}T$ only. The native free energy barrier is subsequently estimated from the equilibrium work required to reach spontaneous nucleation. Active perturbation methods provide an intuitive reaction coordinate, i.e., the distance between two pull groups, which closely mimics how SNARE complexes exert force on the membrane via their hydrophilic C-termini. Additionally, they enable the studying of asymmetric stalk to pore transitions (leaky fusion pathways).

However, because reversal of the perturbation does not reverse the fusion reaction after barrier crossing, the reaction coordinate is not thermodynamically reversible. As a consequence, the apparent equilibrium work to bring the probes in close proximity would vanish in the limit of infinite sampling. Nevertheless, given that simulation protocols are kept consistent, active perturbation methods can provide valuable qualitative insights into trends and the relative dependencies of external factors.

Further, there is often some ambiguity on how and where to exert point-like forces. For example, when examining how the attachment of additional residues to the SNARE C-termini affects the stalk expansion barrier, one obtains different results depending on whether the force is applied to the original C-terminus of the wild type, the new C-terminus, or the center of mass of the whole TMD (Sharma and Lindau 2018; Risselada and Mayer 2020). Simultaneous perturbation of multiple SNARE complexes present at the fusion site will introduce additional inaccuracies.

*Permutation Reduction.* Umbrella sampling of collective modes (e.g., principal components) is a standard technique for probing the free energy for conformational transitions in proteins. Its application to membrane transformations is, however, complicated by the diffusion of the lipids within each leaflet. This problem has been solved recently by the permutation reduction method (Reinhard and Grubmüller 2007; Heinz and Grubmüller 2020), which un-does this diffusion without changing the physics of the system and, hence, allows for the application of Umbrella sampling (Bubnis and Risselada 2016). (Fig. 4b). This method is highly effective when structural changes are characterized by a dominant collective mode such as, for example, bending of a membrane sheet (Bubnis and Risselada 2016). Large collective modes can be alternatively modeled by explicitly using membrane curvature as a collective reaction coordinate (Bouvier 2019). However, smoothly enforcing the structure through a transition state of single molecular nature—such as the formation of a stalk (Risselada et al. 2014)—is hard to achieve by coupling to only a single or even a few collective modes. Achieving thermodynamic reversibility may thus be rather challenging.

*Density-field based methods* Structural transformations in membrane fusion are collective and involve a large amount of highly diffusive molecules. Local densities provide an effective high dimensional reaction coordinate to delineate complex self-assembled structures, because density is (i) indiscriminate for the individual labels which molecular species carry within molecular simulations, but (ii) does discriminate between subtle differences in adopted structure (Müller et al. 2012; Smirnova and Müller 2015; Smirnova et al. 2019; Endter et al. 2020). Within this approach, local densities of a chosen group of atoms or molecules (typically the hydrophobic lipid tails) are defined on a discrete three-dimensional collocation lattice spanning the simulation box (see Fig. 4c). Changes in the configuration are traced locally at each grid point and are used to guide the simulation. The difference between reference and instantaneous local densities are used to calculate restoring forces due to the applied harmonic potential. Forces are subsequently redistributed to each particle according to a differentiable weighting function.

Because the order parameter is high dimensional (the dimension is defined by the number of density latices), it enables a large diversity of different pathways. Consequently, it can be used in conjunction with string methods (Müller et al. 2012; Smirnova et al. 2019). The main idea of such an approach is to describe the most likely reaction path – the minimum free-energy path (MFEP)—by a sequence of membrane configurations (the string) which connects the reactant state and product state (see Fig. 4c). To this aim, an initial sequence of sample configurations (the initial path) is constructed based on simple, linear interpolation between the known densities of the reaction and product state. Each of these sample configurations is studied by a separate, independent MD simulation and the local chemical potential, i.e. the derivative of the free energy with respect to density, is obtained. Knowledge of this chemical potential is exploited to update all sample configurations that comprise the path within an additional but separate pseudodynamical step. This update locally minimizes the free energy, subject to a constraint that the (normalized) distance between neighboring sample configurations along the path remains uniform.

*Engineered potentials.* Potentials with a complex engineered shape can be alternatively used to control the fusion reaction (Kawamoto and Shinoda 2014; Kawamoto et al. 2015). The question is to which extend the obtained results would depend on the parametrization of the potential, because the approach involves local perturbation of the membranes. In contrast to using implicit umbrella potentials, fusion can be alternatively driven by engineered particle assemblies coined ‘Gizmo’ that artificially control the progress of the fusion reaction by enforcing gradual changes of the membrane topology (Bubnis and Grubmüller 2020). This approach yields a thermodynamically reversible pathway, but require some parametrization and fine-tuning (Bubnis and Grubmüller 2020).

## Conclusions and outlook

In summary, fusion proteins can play quite versatile and involved roles during all stages of the fusion reaction. Their roles go far beyond forcing the opposing membranes into close proximity to drive stalk formation and fusion. Early elastic continuum models have laid the necessary foundations in understanding how the elastic properties of lipid membranes can determine the free energy landscape of membrane fusion as well as the prediction of the essential structural intermediates formed during such an reaction. Within this framework, the potential roles of fusion proteins could only be coarsely understood in terms of their effective molecular shape, generated membrane curvature, their effect on the elastic moduli, and the generation of membrane tension (Chernomordik and Kozlov 2005, 2008).

Molecular simulations have played a central role in providing additional detailed molecular insight and understanding of how fusion proteins actively overcome the different free energy barriers of the fusion reaction up to the expansion of the fusion pore. Unexpectedly, molecular simulations have revealed a preference of the biological fusion reaction to proceed through asymmetric pathways resulting in the formation of, e.g., a stalk-hole complex, rim-pore, or vertex pore. Furthermore, force-field based molecular simulations, in particular coarse-grained simulations, are now able to directly resolve the minimum free-energy path in protein-mediated fusion and to quantify the free energies of formed reaction intermediates (Smirnova et al. 2019; Endter et al. 2020; Bubnis and Grubmüller 2020).

Coarse-grained force fields come with their shortcomings and pitfalls, however. Even though the models are parameterized on reproducing thermodynamic properties such as, for example, densities of bulk phases and mixtures (partitioning free energies), the inevitable reduction of entropy in coarse-grained models must be effectively compensated by an enthalpic term. Therefore, resulting differences in entropic and enthalpic contribution can bias the obtained free energy profile. Perhaps most of the bias, however, will simply stem from differences in force-field version. For example, the tails of DOPC lipids are modeled by either four or five tail beads in the new and old version respectively. These different DOPC models form membranes that significantly differ in thickness and concomitant elastic moduli (Marrink et al. 2004, 2007; Bubnis and Risselada 2016). It is rather likely that this will also result in a different free energy of fusion intermediates.

With the arrival of GPUs and fast free energy calculation methods (e.g., Endter et al. 2020) the corresponding free energies of fusion intermediates on an atomistic level have now in principle become accessible. Insight herein will gain valuable information on the predictive value of coarse-grained models of coarse-grained simulations and will result in further improvements of their parameterization. Consequently, direct comparisons with experiments such as the predicted hemifusion and fusion rates of small liposomes (François-Martin et al. 2017) will become possible in the near future. However, a quantitative comparison between simulation and experiments is still hindered by the fact that simulations typically can not include all of the relevant free energy barriers, but rather focus on individual steps—for example, modeling a scenario where the membranes are already brought in close proximity by a partially assembled SNARE complex. Therefore, experimental knowledge of those individual barriers must be used for the simulations as input. In this respect, (hidden) Markov state modeling on experimental data of fusion kinetics offers a promising route. Furthermore, albeit more qualitative, validation and especially the prediction of putative point mutations also will provide a direct and discriminate way to validate the reaction pathways resolved in molecular simulations.

In principle, the fusion process between a realistic model of a 40 nm-sized synaptic vesicle (Takamori 2006) and the presynaptic membrane, including all the associated proteins (Fig. 1a), is already within reach with current computational equipment and software. In such a case, the simulation setup and positioning of the individual proteins can benefit from insights from cryo electron microscopy (EM) observations and resolved protein-protein interactions. Since the structure and stoichiometry of the participating proteins are largely known, optimal density overlap between simulations and cryo-EM data could be achieved using molecular dynamics simulations driven by bias potentials derived from correlations between the simulated structure and the cryo-EM density (Igaev et al. 2019), or rigid-body Monte-Carlo simulations (fixing the protein’s internal degrees of freedom) (Dodd et al. 2020).

The questions of interest that can be addressed using such an approach is to which extend the membrane proteins and the concomitant free energy barriers of the fusion reaction are affected by the (extreme) abundance of proteins. Indeed, membrane proteins occupy about 30% of the total surface area biological membranes (Dupuy and Engelman 2008). In contrast, the free energy landscape of fusion has been traditionally understood from the behavior and elastic properties of pure lipid membranes. Another relevant question is how protein crowding in both the plasma membrane and synaptic vesicle enables a close contact between the membrane fusing membranes. In fact, the initial clearance of space at the site of membrane fusion may impose a significant additional free energy barrier within the fusion reaction. It is also possible that some degree of domain formation, which clears out membrane proteins, is essential for fusion to occur. If fusion occurs at domains, the line tension associated with their interface may contribute to the contraction of fusion pores (invagination) similar to its role in domain facilitated membrane budding (Lipowsky 1992).

Finally, realistic simulation of viral fusion such as, for example, the Influenza virus is equally possible within the current computational capabilities, at least on a detailed coarse-grained level (Reddy et al. 2015). A remaining complication in simulating viral fusion, however, is the effect of local pH on the structure of viral proteins such as, the fusion loops and matrix proteins, since lowering of the pH plays a predominant role in facilitating the fusion reaction (Lee 2010). The effect of pH, which can significantly change because of local electrostatic interactions, is not trivially incorporated in coarse-grained nor atomistic simulations. However, pioneering methodological advances are on the way that enable simulation of constant pH on an atomistic level (see, e.g., Refs.(Mongan and Case 2005; Donnini et al. 2011; Dobrev et al. 2020)). Future methodological developments will also focus on how to additionally include configurational changes and dynamics of the fusion proteins themselves (e.g., the assembly of the SNAREs complex) within the already quite high-dimensional reaction coordinates, thereby gaining an even more complete and direct molecular picture on how fusion proteins work.
